# Draft Genome Sequences of *Sulfurovum* spp. TSL1 and TSL6, Two Sulfur-Oxidizing Bacteria Isolated from Marine Sediment

**DOI:** 10.1128/MRA.00922-21

**Published:** 2022-01-06

**Authors:** Yong Guo, Hideyuki Ihara, Tomo Aoyagi, Tomoyuki Hori, Yoko Katayama

**Affiliations:** a Environmental Management Research Institute, National Institute of Advanced Industrial Science and Technology (AIST), Tsukuba, Japan; b Institute of Agriculture, Tokyo University of Agriculture and Technology, Fuchu, Tokyo, Japan; c Tokyo National Research Institute for Cultural Properties, Taito-ku, Tokyo, Japan; Montana State University

## Abstract

*Sulfurovum* spp. TSL1 and TSL6 are sulfur-oxidizing chemolithoautotrophic bacteria isolated from the tsunami-launched marine sediment in the Great East Japan earthquake. This announcement describes the draft genome sequences of the two isolates that possess the gene sets for the sulfur oxidation pathway.

## ANNOUNCEMENT

**FIG 1:**
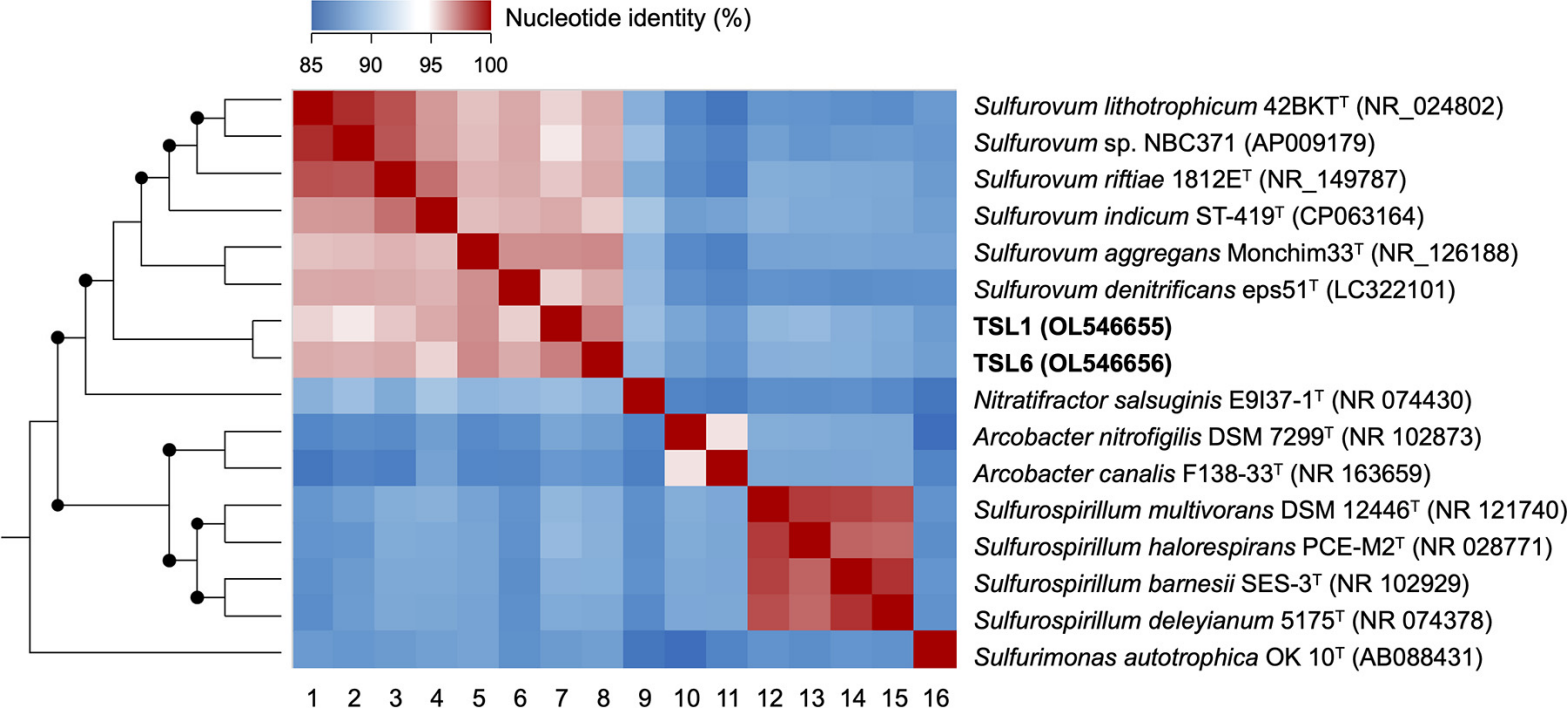
Phylogenetic relationship of TSL1 and TSL6 with the representative strains in the genera *Sulfurovum*, *Nitratifractor*, *Arcobacter*, *Sulfurospirillum*, and *Sulfurimonas*. Neighbor-joining phylogenetic tree (left) is reconstructed using near full-length 16S rRNA gene sequences, and bootstrap values (1,000 replicates) of more than 70% are indicated by solid circles at the branches. Heatmap (right) shows pairwise nucleotide identities of the near full-length 16S rRNA gene sequences. Column numbers below the heatmap indicate the bacterial strains as follows: 1, Sulfurovum lithotrophicum 42BKT^T^; 2, *Sulfurovum* sp. strain NBC371; 3, Sulfurovum riftiae 1812E^T^; 4, Sulfurovum indicum ST-419^T^; 5, Sulfurovum aggregans Monchim33^T^; 6, Sulfurovum denitrificans eps51^T^; 7, TLS1; 8, TSL6; 9, Nitratifractor salsuginis E9I37-1^T^; 10, Arcobacter nitrofigilis DSM 7299^T^; 11, Arcobacter canalis F138-33^T^; 12, Sulfurospirillum multivorans DSM 12446^T^; 13, Sulfurospirillum halorespirans PCE-M2^T^; 14, Sulfurospirillum barnesii SES-3^T^; 15, Sulfurospirillum deleyianum 5175^T^; and 16, Sulfurimonas autotrophica OK 10^T^. TSL1 and TSL6 shared 95.2% to 96.8% of the 16S rRNA gene identities with the type strains of type species in the genus *Sulfurovum*, indicating that the two strains can represent the undescribed species affiliated in the genus *Sulfurovum*.

An epsilonproteobacterial genus, *Sulfurovum*, mainly inhabiting deep-sea hydrothermal vents, is a group of sulfur-oxidizing chemolithoautotrophs governing the biogeochemical cycling of elements in seafloor ecosystems (1–5). A large amount of marine sediment was launched on land by a tsunami that occurred in the Great East Japan earthquake, and thereafter, the *Sulfurovum*-affiliated bacteria participated in sulfur oxidation when the marine sediment was exposed to oxic conditions (6). Two sulfur-oxidizing bacteria, TSL1 and TSL6, were isolated from the marine sediment (38°25′N, 141°14′E; Higashimatsushima, Miyagi, Japan). Briefly, the sediment was preincubated aerobically for 3 days and then incubated using serial dilution in basal salts of MJ medium (7) supplemented with a vitamin solution containing biotin, folic acid, pyridoxine hydrochloride, thiamine hydrochloride, riboflavin, nicotinic acid, DL-calcium pantothenate, vitamin B_12_, *p*-aminobenzoic acid, and lipoic acid (8), and thiosulfate or elemental sulfur as an energy source. After repeating serial subcultivation 4 to 7 times, TSL1 and TSL6 were obtained as pure cultures using the thiosulfate and elemental sulfur as energy source, respectively. Based on 16S rRNA gene sequences (Fig. 1), these two bacteria could represent the undescribed *Sulfurovum* species. Here, we sequenced the genomes of these bacteria to reveal the genetic features underlying their biogeochemical activities.

TSL1 and TSL6 were aerobically grown in the above-mentioned medium supplemented with the vitamin solution, PIPES [piperazine-*N*,*N*′-bis(2-ethanesulfonic acid)] (20 mM), and sodium thiosulfate (20 mM) at room temperature for 2 to 3 weeks. Genomic DNA was isolated using a lysozyme buffer method (9). Paired-end libraries (insert size, ∼350 bp) were prepared using a VAHTS universal DNA library prep kit for Illumina (Vazyme Biotech) and sequenced using a HiSeq 2500 system (Illumina), which generated 8.9 and 11.1 million reads (2 × 150 bp) for TSL1 and TSL6, respectively. Adapters were removed using Cutadapt v3.1 (10), while the reads with a Q score of <30 were removed using Sickle v1.33 (https://github.com/najoshi/sickle). High-quality reads were preassembled using Unicycler v0.4.8 with default settings (11) and then assembled using SPAdes v3.15.0 (12) with the trusted-contigs tool, as previously described (13). The draft genomes were annotated by the DFAST v1.4.0 (14) and KofamKOALA v2021-07-05 (15). Whole-genome average nucleotide identity (ANI) and digital DNA-DNA hybridization (dDDH) between the two genomes were calculated using the JSpeciesWS (16) and GGDC v2.1 (17), respectively.

TSL1 had a genome size of 2,353,154 bp assembled into 6 contigs (*N*_50_ value, 1,782,176 bp; GC content, 40.7 mol%; coverage, 500×), while TSL6 had the genome size of 2,254,663 bp assembled into 7 contigs (*N*_50_ value, 760,548 bp; GC content, 38.4 mol%; coverage, 650×) (Table 1). A total of 2,359 genes were predicted as 3 rRNAs, 42 tRNAs, and 2,314 protein-coding sequences (CDSs) in the TSL1 genome, while there was a total of 2,259 genes as 3 rRNAs, 43 tRNAs, and 2,213 CDSs in the TSL6 genome (Table 1). The ANI and dDDH values between the two isolates were calculated to be 86.5% (aligned coverage, 75.9%) and 32.3%, respectively, which suggests that TSL1 and TSL6 can be classified into different species. Genome annotation revealed that both of the two bacteria possessed a sulfur oxidation system, which would be helpful to understand their roles in the acidification of the tsunami-launched marine sediment (6).

**TABLE 1:** Statistics for genome assemblies of *Sulfurovum* spp. TSL1 and TSL6

Characteristic	Data for:	
	TSL1	TSL6
No. of contigs	6	7
*N*_50_ value (bp)	1,782,176	760,548
GC content (mol%)	40.7	38.4
Total length (bp)	2,353,154	2,254,663
Genome coverage (×)	500	650
Genome completeness (%)	99.6	99.6
No. of rRNAs	3	3
No. of tRNAs	42	43
No. of CDSs	2,314	2,213
GenBank accession no.	BPFI01000001 to BPFI01000006	BPFJ01000001 to BPFJ01000007
DRA accession no.	DRA011857	DRA011856

### Data availability.

The draft genome sequences of *Sulfurovum* spp. TSL1 and TSL6 are available under the DDBJ/ENA/GenBank accession numbers BPFI01000001 to BPFI01000006 and BPFJ01000001 to BPFJ01000007, respectively. The raw reads are available under the DRA accession numbers DRA011857 and DRA011856 for TSL1 and TSL6, respectively.

## References

[1] Inagaki F, Takai K, Nealson KH, Horikoshi K. 2004. *Sulfurovum lithotrophicum* gen. nov., sp. nov., a novel sulfur-oxidizing chemolithoautrotroph within the *ε-Proteobacteria* isolated from Okinawa Trough hydrothermal sediments. Int J Syst Evol Microbiol 54:1477–1482. doi:10.1099/ijs.0.03042-0. 15388698

[2] Mino S, Kudo H, Arai T, Sawabe T, Takai K, Nakagawa S. 2014. *Sulfurovum aggregans* sp. nov., a hydrogen-oxidizing, thiosulfate-reducing chemolithoautotroph within the *Epsilonproteobacteria* isolated from a deep-sea hydrothermal vent chimney, and an emended description of the genus *Sulfurovum*. Int J Syst Evol Microbiol 64:3195–3201. doi:10.1099/ijs.0.065094-0. 24966202

[3] Giovannelli D, Chung M, Staley J, Starovoytov V, Bris NL, Vetriani C. 2016. *Sulfurovum riftiae* sp. nov., a mesophilic, thiosulfate-oxidizing, nitrate-reducing chemolithoautotrophic epsilonproteobacterium isolated from the tube of the deep-sea hydrothermal vent polychaete *Riftia pachyptila*. Int J Syst Evol Microbiol 66:2691–2701. doi:10.1099/ijsem.0.001106. 27116914

[4] Mori K, Yamaguchi K, Hanada S. 2018. *Sulfurovum denitrificans* sp. nov., an obligately chemolithoautotrophic sulfur-oxidizing epsilonproteobacterium isolated from a hydrothermal field. Int J Syst Evol Microbiol 68:2183–2187. doi:10.1099/ijsem.0.002803. 29757127

[5] Xie S, Wang S, Li D, Shao Z, Lai Q, Wang Y, Wei M, Han X, Jiang L. 2021. *Sulfurovum indicum* sp. nov., a novel hydrogen- and sulfur-oxidizing chemolithoautotroph isolated from a deep-sea hydrothermal plume in the Northwestern Indian Ocean. Int J Syst Evol Microbiol 71:e004748. doi:10.1099/ijsem.0.004748. 33734956

[6] Ihara H, Hori T, Aoyagi T, Takasaki M, Katayama Y. 2017. Sulfur-oxidizing bacteria mediate microbial community succession and element cycling in launched marine sediment. Front Microbiol 8:152. doi:10.3389/fmicb.2017.00152. 28217124 PMC5289976

[7] Sako Y, Takai K, Ishida Y, Uchida A, Katayama Y. 1996. *Rhodothermus obamensis* sp. nov., a modern lineage of extremely thermophilic marine bacteria. Int J Syst Bacteriol 46:1099–1104. doi:10.1099/00207713-46-4-1099. 8863442

[8] Balch WE, Fox GE, Magrum LJ, Woese CR, Wolfe RS. 1979. Methanogens: reevaluation of a unique biological group. Microbiol Rev 43:260–296. doi:10.1128/mr.43.2.260-296.1979. 390357 PMC281474

[9] Guo Y, Takashima Y, Sato Y, Narisawa K, Ohta H, Nishizawa T. 2020. *Mycoavidus* sp. strain B2-EB: comparative genomics reveals minimal genomic features required by a cultivable *Burkholderiaceae-*related endofungal bacterium. Appl Environ Microbiol 86:e01018-20. doi:10.1128/AEM.01018-20. 32651207 PMC7480390

[10] Martin M. 2011. Cutadapt removes adapter sequences from high-throughput sequencing reads. EMBnet J 17:10–12. doi:10.14806/ej.17.1.200.

[11] Wick RR, Judd LM, Gorrie CL, Holt KE. 2017. Unicycler: resolving bacterial genome assemblies from short and long sequencing reads. PLoS Comput Biol 13:e1005595. doi:10.1371/journal.pcbi.1005595. 28594827 PMC5481147

[12] Bankevich A, Nurk S, Antipov D, Gurevich AA, Dvorkin M, Kulikov AS, Lesin VM, Nikolenko SI, Pham S, Prjibelski AD, Pyshkin AV, Sirotkin AV, Vyahhi N, Tesler G, Alekseyev MA, Pevzner PA. 2012. SPAdes: a new genome assembly algorithm and its applications to single-cell sequencing. J Comput Biol 19:455–477. doi:10.1089/cmb.2012.0021. 22506599 PMC3342519

[13] Guo Y, Aoyagi T, Inaba T, Sato Y, Habe H, Hori T. 2020. Complete genome sequence of *Desulfuromonas* sp. strain AOP6, an iron(III) reducer isolated from subseafloor sediment. Microbiol Resour Announc 9:e01325-19. doi:10.1128/MRA.01325-19. 32193240 PMC7082459

[14] Tanizawa Y, Fujisawa T, Nakamura Y. 2018. DFAST: a flexible prokaryotic genome annotation pipeline for faster genome publication. Bioinformatics 34:1037–1039. doi:10.1093/bioinformatics/btx713. 29106469 PMC5860143

[15] Aramaki T, Blanc-Mathieu R, Endo H, Ohkubo K, Kanehisa M, Goto S, Ogata H. 2020. KofamKOALA: KEGG Ortholog assignment based on profile HMM and adaptive score threshold. Bioinformatics 36:2251–2252. doi:10.1093/bioinformatics/btz859. 31742321 PMC7141845

[16] Richter M, Rosselló-Móra R, Glöckner FO, Peplies J. 2016. JSpeciesWS: a web server for prokaryotic species circumscription based on pairwise genome comparison. Bioinformatics 32:929–931. doi:10.1093/bioinformatics/btv681. 26576653 PMC5939971

[17] Meier-Kolthoff JP, Auch AF, Klenk HP, Göker M. 2013. Genome sequence-based species delimitation with confidence intervals and improved distance functions. BMC Bioinformatics 14:60. doi:10.1186/1471-2105-14-60. 23432962 PMC3665452

